# Phylogenomic and single nucleotide polymorphism analyses revealed the hybrid origin of *Spondias bahiensis* (family Anacardiaceae): d*e novo* genome sequencing and comparative genomics

**DOI:** 10.1590/1678-4685-GMB-2017-0256

**Published:** 2018-11-29

**Authors:** Lydayanne Lilás de Melo Nobre, José Daniel Oliveira dos Santos, Rychard Leite, Cícero Almeida

**Affiliations:** ^1^ Genetic Resources Laboratory, Arapiraca Campus, Universidade Federal de Alagoas, Arapiraca, AL, Brazil.

**Keywords:** rDNA, phylogenetics, evolution, hybrid offspring

## Abstract

The genus *Spondias* (family Anacardiaceae) comprises 19 taxa, ten of which occur in Neotropical regions. *Spondias bahiensis* has been suggested to be a hybrid, although initial evidence does not support this hypothesis. The aim of this study was to test the hypothesis of the hybrid origin of *S. bahiensis* using high-throughput sequencing with single nucleotide polymorphism (SNP) analysis, characterization of intragenomic nuclear ribosomal DNA (nrDNA), and nuclear and chloroplast phylogenomic analyses. The SNP analysis revealed a high number of SNPs in the *S. bahiensis* genome, and with respect to nrDNA, *S. bahiensis* shared approximately half of the SNP alleles with *S. tuberosa*, but not with *S. mombin*. Combining the SNP analysis with nrDNA phylogeny confirmed the hybrid origin of *S. bahiensis* and put *S. tuberosa* as the female genitor. Considering the phylogeny of the genus *Spondias* and intraspecific SNPs in *S. bahiensis*, the putative male genitor is *S. dulcis*.

## Introduction

The genus *Spondias* (family Anacardiaceae) comprises 19 taxa, 10 of which occur in Neotropical regions (Mitchell and Daly, 2015). *Spondias tuberosa* Arruda, *S. mombin* L., and *S. purpurea* L. are widespread most notably in northeastern Brazil, in addition to a new species (*Spondias bahiensis* P. Carvalho, van den Berg & M. Machado), which was recently described by Machado *et al.* (2015). *Spondias tuberosa*, *S. mombin* and *S. bahiensis*, which are known by the vernacular names umbu, cajá, taperebá, and umbu*-*cajá, occur in northeastern Brazil. *Spondias bahiensis* has been popularly known as a hybrid between *S. tuberosa* and *S. mombin*, but this hypothesis was contested by chromosome banding and genomic *in situ* hybridization (GISH) studies (Almeida *et al.*, 2007), DNA barcoding (Silva *et al.*, 2015), and molecular and morphological analyses (Machado *et al.*, 2015). Machado *et al.* (2015) discussed the possibility that *S. bahiensis* could have originated by hybridization between *S. tuberosa* and *S. venulosa*, but there is no evidence in support of this hypothesis either. In this context, a recent study used high-throughput sequencing data to investigate the origin of *S. bahiensis* and the evolution of the genus *Spondias*.

Molecular phylogenetic analysis is essential to elucidate the ecological, evolutionary, and taxonomic characteristics of organisms. To date, most phylogenetic methods have used only a tiny portion of the nuclear and chloroplast genomes, generally the phylogeny of genes or intergenic spacers. With next generation sequencing technologies, whole genome sequence data have become faster and cheaper to obtain, and phylogenetic reconstruction has dramatically accelerated, for instance, high-throughput sequencing has facilitated phylogenomic studies based on multilocus phylogeny (Comer *et al.*, 2015), repetitive DNA (satellites and mobile elements) (Macas *et al.*, 2015), chloroplast genomic DNA (Barrett *et al.*, 2016), and nuclear ribosomal DNA (nrDNA) (Weitemier *et al.*, 2015). Although nrDNA is not suitable for phylogenetic studies because the copies are assembled as tandem repeats and homologous recombination occurs frequently, nrDNA alleles may become species-specific, thereby allowing hybrid detection. Single nucleotide polymorphism (SNP) analysis has been utilized in studies of genomic characterization and diversity, but heretofore the SNP approach has not been used for genomic analysis of hybrids. The aim of the present study was to determine the hybrid origin of *S. bahiensis* using high-throughput sequencing to analyze SNP variants, nrDNA alleles, and the nuclear and chloroplast phylogenomics.

## Material and Methods

### High-throughput sequence data

Samples of *S. tuberosa*, *S. mombin*, and *S. bahiensis* were collected in the state of Alagoas, Brazil, and DNA extraction was performed from leaves using the cetyl trimethylammonium bromide extraction method, as described by Doyle and Doyle (1987). The quality and quantity of the extracted DNA were verified by visualization on a 1% agarose gel and spectrophotometry, respectively. Species identification was performed using the DNA barcodes described by Silva *et al.* (2015). DNA samples were fragmented into 400–500 bp to construct a sequencing library. The fragments were ligated with adapters using the Nextera DNA Sample Preparation kit (Illumina, Inc., San Diego, CA, USA). Sequencing of 100-nt single-end reads for *S. bahiensis* and 100-nt paired-end reads for *S. tuberosa* and *S. mombin* was performed using the Illumina HiSeq2500 platform at the Central Laboratory for High Performance Technologies in Life Sciences (LaCTAD) of the State University of Campinas (São Paulo, Brazil). For *Pistacia vera* (utilized as outgroup), sequence read archive (SRA) files were unpacked into FASTQ using the FASTQ-DUMP executable function from the SRA toolkit. FASTQ files were then filtered with a minimum quality of 10 and converted to FASTA files.

### Phylogenomic analysis

Chloroplast genome sequences were obtained from the National Center for Biotechnology Information database (https://www.ncbi.nlm.nih.gov/) and aligned using the MAFFT v7.017 algorithm (Katoh and Standley, 2013) implemented as the “Multiple align” tool in Geneious R9 (http://www.geneious.com). Bayesian analysis was performed using Beast v2 (Drummond and Rambaut, 2007) and posterior distribution was approximated using the Markov chain Monte Carlo (MCMC) method with 10 million steps. Convergence of the parameters was checked using the Tracer 1.5 program (Rambaut *et al.*, 2018). For nuclear phylogenomic analysis, the phylogenies of the reads were reconstructed using the assembly and alignment-free (AAF) method (Fan *et al.*, 2015), and phylogenetic relationships were estimated using high-throughput sequencing data from the whole genomes. The phylogenies were reconstructed directly from unassembled genome sequence data.

### Discovery and analysis of SNPs

For SNP analysis, the *de novo* contigs were constructed from 198 million reads from *S. tuberosa* using Ray software (Boisvert *et al.*, 2012), with a minimum size of 200 nt, 31 k-mers, and 8 coverage. The largest contigs were then used as references. The data from *S. tuberosa*, *S. bahiensis*, and *S. mombin* were aligned for SNP identification using GATK software (Van der Auwera *et al.*, 2013). The results, in vcf format, were analyzed using R package (vcfR packager) (Knaus and Grunwald*,* 2017).

#### Nuclear ribosomal analysis

The paired-end reads from *S. mombin* were utilized for repeat analysis, which was performed with Tandem Repeat Analyzer (TAREAN) software (Novák *et al.*, 2017) for repeat identification. TAREAN is a computational pipeline for identification of repeats from unassembled sequence reads. After identification of the nrDNA, the spacer regions ITS1 and ITS2 were utilized for allele identification by sequence mapping. A phylogenetic tree of the alleles was constructed for characterization of intragenomic nrDNA polymorphisms. The sequences were aligned using the MAFFT v7.017 program (Katoh and Standley, 2013) implemented as the “Multiple align” tool in Geneious R9 (http://www.geneious.com). Bayesian analysis was performed using Beast v2 (Drummond and Rambaut, 2007) and posterior distribution was approximated using the MCMC method with 10 million steps. The convergence of the parameters was checked using the Tracer 1.5 program (Rambaut *et al.*, 2018). Cluster analysis by similarity-based clustering of Illumina reads was performed using RepeatExplorer (Novák *et al.*, 2010) individually for ITS1 and ITS2.

### Results

#### Phylogenomic relationships

**Figure 1:**
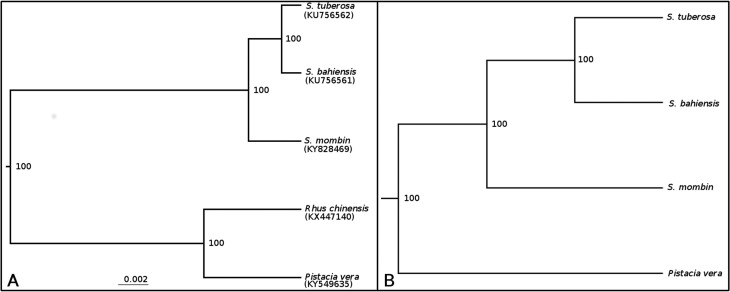
Phylogenetic analysis of the chloroplast and nuclear genomes. (A) Phylogenetic relationships using the Bayesian approach of the complete chloroplast genomes of the genus *Spondias* and out group *Rhus chinenesis* and *Pistacia vera*. The support values are estimated with posterior probabilities (in percentages). (B) A phylogenetic tree reconstructed with the AAF approach using nuclear reads. The support values are estimated with bootstrap values (in percentages).

The phylogenomic analysis using complete chloroplast genomes (Figure 1A) and nuclear data obtained by the AAF approach (Figure 1B) revealed three clades: one formed by *S. tuberosa* and *S. bahiensis*, an intermediary clade consisting of *S. mombin*, and an out-group clade formed by *Pistacia vera* and *Rhus chinensis* for the chloroplast tree and *Pistacia vera* for the nuclear phylogenomic tree*.* Pairwise comparisons for chloroplast analyses identified 856 SNPs between *S. tuberosa* and *S. bahiensis*, 3042 between *S. tuberosa* and *S. mombin*, and 3292 between S*. bahiensis* and *S. mombin.*

#### SNP analysis

**Figure 2:**
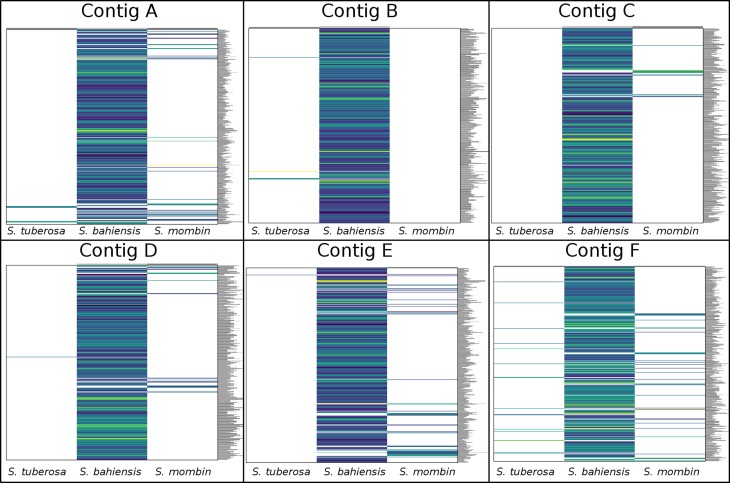
SNP distribution and density of six contigs in *Spondias tuberosa, Spondias bahiensis*, and *Spondias mombin*.

**Figure 3:**
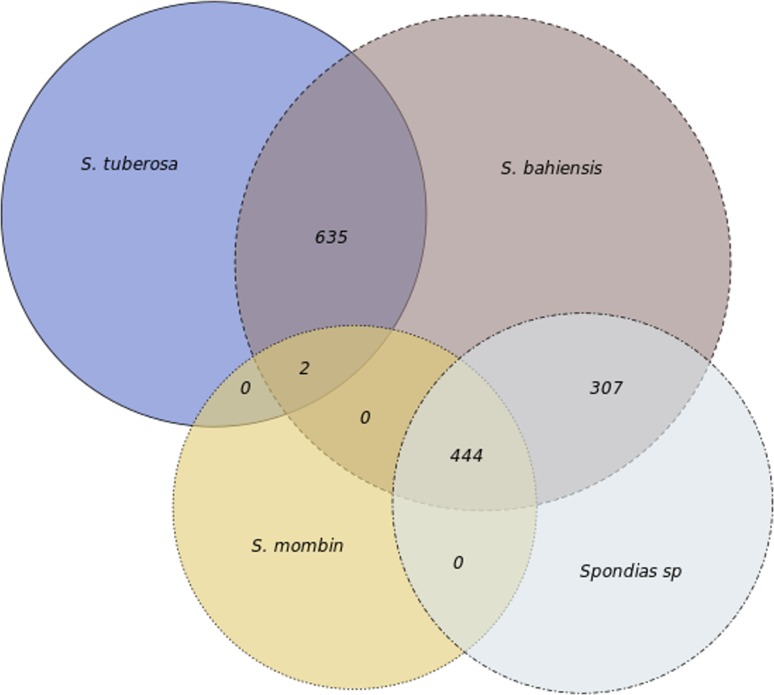
Venn diagram of the allelic distribution of *Spondias bahiensis*. The Venn diagram shows the number of alleles that are shared among *Spondias bahiensis*, *Spondias tuberosa*, and *Spondias mombin*.

For SNP discovery, six contigs, ranging in length from 48,859 to 54,663 bp, were analyzed with GATK software. The results revealed 0–31 SNPs in *S. tuberosa* and 0–48 in *S. mombin*, with a mean of two and five SNPs for each 10 kb (Table 1 and Figure 2). Remarkably, *S. bahiensis* showed the highest SNP content of 678–936 SNPs, with a mean of 166 SNPs for each 10 kb. The detailed analysis for contig F showed that *S. bahiensis* shared only half of the alleles with *S. tuberosa*, indicating that *S. tuberosa* is a genitor of *S. bahiensis* (Figure 3).

**Table 1:** Number of SNPs present in six contigs for *Spondias tuberosa*, *Spondias bahiensis*, and *Spondias mombin*.

		Intra-specific			
Contig	Length (bp)	*S. tuberosa*	*S. bahiensis*	*S. mombin*	Inter-specific^*^
Contig A	54,663	24	936	39	946
Contig B	51,123	6	892	0	621
Contig C	49,173	0	838	13	778
Contig D	47,249	1	828	18	676
Contig E	50,619	1	834	48	821
Contig F	48,859	31	678	30	835
Total	301,686	66	5,006	148	4,677

^*^ Variation between *S. tuberosa* and *S. mombin.*

#### Nuclear ribosomal phylogeny

**Figure 4:**
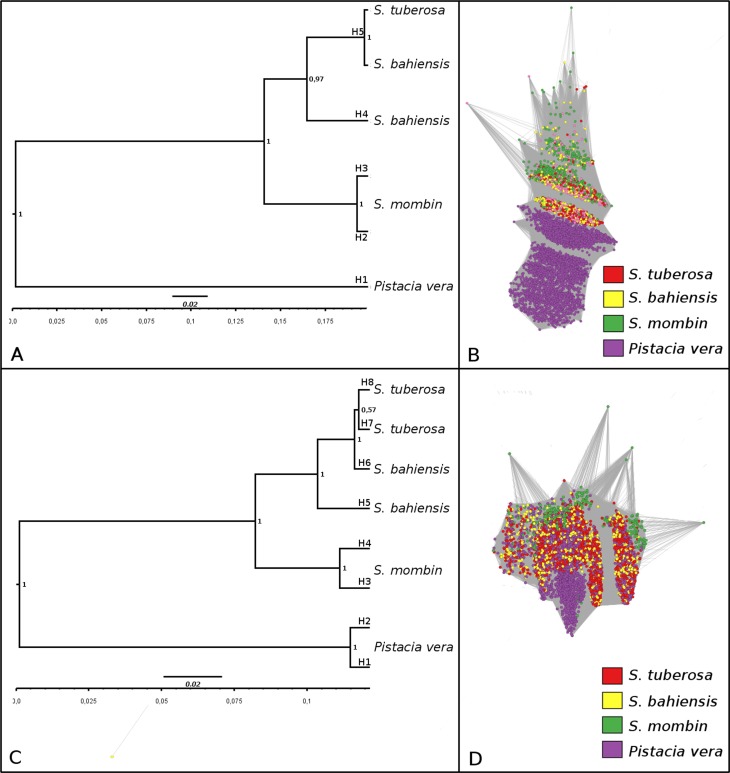
Phylogenetic analysis of ITS1 and ITS2. Phylogenetic relationships between ITS1 (A) and ITS2 (C) using the Bayesian approach. Graphic representation of ITS1 (B) and ITS2 (D), where reads from the species are highlighted in red (*Spondias tuberosa*), yellow (*Spondias bahiensis*), green (*Spondias mombin*), and purple (*Pistacia vera*).

The allele identification for ITS1 and ITS2 of *Spondias* and *Pistacia* revealed five alleles for ITS1 (H1–H5) (Figure 4A) and eight for ITS2 (H1–H8) (Figure 4C). Among the ITS1 alleles, H1 was exclusive to *P. vera* (outgroup), H2 and H3 to *S. mombin*, H4 to *S. bahiensis*, and H5 to both *S. tuberosa* and *S. bahiensis*, indicating that *S. tuberosa* and *S. bahieneis* shared the same alleles. The intraspecific variation observed between H2 and H3 for ITS1 (*S. mombin*) was limited to only one SNP.

For ITS2, the phylogenetic relationships showed the same topology. *S. tuberosa* and *S. bahiensis* showed a high relationship with allele differences of only one SNP (alleles H6 and H7). Intraspecific variation was observed in *S. tuberosa* and *S. mombin*, with one and two SNPs, respectively. Similar to ITS1, ITS2 analyses also suggested that *S. tuberosa* is the genitor of *S*. *bahiensis*. Graph analysis of ITS1 (Figure 4B) and ITS2 (Figure 4D) using reads from both species showed clustering of dots corresponding to the specific species. Notably, reads from *S. tuberosa* and *S. bahiensis* were overlapping, while reads from *S. mombin* were not overlapping, suggesting that *S. tuberosa* and *S. bahiensis* are closely related.

### Discussion

The phylogenetic relationships in the genus *Spondias* have been supported by molecular data (Silva *et al.*, 2015; Machado *et al.*, 2015) and cytogenetic data (Almeida *et al.*, 2007). These studies discuss a possible hybrid origin of a plant popularly known “umbu-cajá” from *S. tuberosa* and *S. mombin*. This was not supported by chromosome banding or GISH studies (Almeida *et al.*, 2007). Furthermore, phylogenetic studies of chloroplast regions and expressed sequence tags (Machado *et al.*, 2015; Silva *et al.*, 2015) suggested that umbu-cajá is a distinct species, which was named *S. bahiensis* by Machado *et al.* (2015).

Here, a combination of different approaches was utilized to assess the hybrid origin of *S. bahiensis*. The approaches included the identification and analysis of the ITS1 and ITS2 alleles, phylogenomic analysis of the complete chloroplast and nuclear genomes, and exhaustive SNP analysis. This is the first study to use nrDNA alleles and SNP approaches to identify the genomic sequence of a hybrid. The results of this study demonstrated the validity of these approaches. Usually, GISH is utilized to distinguish cellular genomes and it has been an important tool for molecular cytogenetics (Silva and Souza, 2013; Ramzan *et al.*, 2017). However, GISH of the genus *Spondias*, using labeled total DNA of *S. mombin* or *S. tuberosa* as a probe and hybridized on metaphase chromosomes of *S. bahiensis*, revealed similar results between the probes, suggesting that there was high identity between the genomes. In this context, the present study showed that SNP analysis is effective for detecting differences in the genomes of naturally occurring hybrids.

The phylogenomic analysis results corroborated the findings of previous studies (Machado *et al.*, 2015; Silva *et al.*, 2015) that showed *S. bahiensis* as genetically intermediate between *S. tuberosa* and *S. mombin*, suggesting a hybrid origin. Pairwise comparisons of the chloroplast genomes showed high identity between *S. tuberosa* and *S. bahiensis*, indicating *S. tuberosa* as the female genitor of the putative hybrid *S. bahiensis* (in Anacardiaceae, the chloroplast DNA is inherited only from the female genitor). The hybrid origin of *S. bahiensis* was confirmed by SNP analysis, which revealed a high number of SNPs in the contigs, with 75.8 and 33.8 more SNPs for *S*. *bahiensis* than for *S. tuberosa* and *S. mombin*, respectively. Analysis of the ITS alleles showed that *S. bahiensis* shared identical or nearly identical alleles with *S. tuberosa*, suggesting *S. tuberosa* as a genitor. The results furthermore indicate that *S. mombin* is not a genitor, as *S. mombin* did not share alleles with *S. bahiensis*. These conclusions are in agreement with the findings of the SNP analyses, showing that half of the alleles of *S. bahiensis* originated from *S. tuberosa*, while the other half were not exclusive to *S. mombin*, suggesting that *S. mombin* is not a genitor of *S. bahiensis*. Because the fruits of *S. tuberosa*, *S. mombin*, and *S. bahiensis* are consumed in Brazil, this conclusion has practical implications, as it helps to stabilize the germplasm banks and breeding strategies for these three species. Notably, the genus *Spondias* is propagated by seeds and vegetative means (Espíndola *et al.*, 2004) while *S. bahiensis* must be propagated vegetatively, as the hybrid origin leads to segregation among progenies.
